# Serum metabolic biomarkers associated with self-reported chronic rhinosinusitis: a population-based cross-sectional study from the KNHANES

**DOI:** 10.1016/j.bjorl.2026.101838

**Published:** 2026-05-28

**Authors:** Han Chen, Lin Wang, Jisheng Zhang, Xudong Yan, Longgang Yu, Yan Jiang

**Affiliations:** a The Affiliated Hospital of Qingdao University, Department of Otolaryngology Head and Neck Surgery, Qingdao Shandong, China; b Fuzhou University Affiliated Provincial Hospital, Fujian Provincial Hospital, Department of Otolaryngology Head and Neck Surgery, Fuzhou, Fujian, China

**Keywords:** Metabolism, Chronic rhinosinusitis, Population-based survey, Uric acid

## Abstract

**Objectives:**

Chronic Rhinosinusitis (CRS) is a prevalent condition with complex pathophysiology. While metabolic factors have been suggested to play a role in CRS, the relationship between serum metabolic markers and CRS prevalence remains unclear.

**Methods:**

This cross-sectional study used data from the 2022 Korean National Health and Nutrition Examination Survey to assess the relationship between serum lipid profiles, glucose metabolism, purine metabolism, and CRS prevalence. A total of 4,759 participants with complete data were included. CRS diagnosis was determined based on self-reported information obtained during health interviews. Serum metabolic markers were measured from fasting blood samples. Logistic regression analyses were conducted to calculate Odds Ratios (OR) and 95% Confidence Intervals (95% CI), while controlling for potential confounding factors.

**Results:**

After controlling for confounders, high serum Uric Acid (UA) levels were significantly associated with a higher risk of CRS (OR = 1.558, 95% CI 1.040, 2.332, p = 0.020). Subgroup analysis revealed a stronger correlation among urban residents (OR = 1.635, 95% CI 1.085, 2.463, p = 0.019). However, no marked association was identified between serum indices of lipid metabolism and glucose metabolism and the prevalence of CRS.

**Conclusion:**

This study found that higher serum UA levels were associated with the presence of CRS, suggesting a potential link between metabolic dysregulation and CRS susceptibility. Further longitudinal and experimental studies are needed to confirm these associations and clarify the underlying mechanisms.

**Level of evidence:**

Level 4 (Cross-sectional study).

## Highlights

• Identified high serum uric acid as a risk factor for chronic sinusitis.
• Association observed in individuals with higher education and urban residents.
• No significant link found between lipid or glucose metabolism and sinusitis risk.
• Study underscores potential role of metabolic dysregulation in CRS pathogenesis.

## Introduction

Chronic Rhinosinusitis (CRS) is a widespread inflammatory condition affecting the nasal and paranasal sinuses, defined by symptoms including rhinorrhea, nasal obstruction, facial pain, and hyposmia lasting for over three months, along with objective signs of nasal polyps, purulent discharge, or radiological inflammation.^1^ CRS severely diminishes patients’ quality of life and poses a significant public health burden, with a global prevalence ranging from 5.5% to 28%.^2^^,^^3^ Although CRS is clinically heterogeneous, its underlying biological mechanisms remain incompletely understood. Increasing evidence suggests that inflammatory and metabolic processes may be associated with CRS. Transcriptomic and proteomic studies, predominantly conducted in tissue samples from patients with Chronic Rhinosinusitis with Nasal Polyps (CRSwNP), have identified alterations in inflammatory mediators and metabolic pathways, providing insights into disease heterogeneity rather than definitive mechanisms applicable to all CRS phenotypes.^4^^,^^5^ These findings underscore the complexity of CRS biology while highlighting the need for population-level evidence.

Previous studies have indicated that metabolic dysregulation may be linked to CRS, particularly in CRSwNP.^6^ Metabolomic analyses of airway inflammatory diseases have revealed changes in lipid, glucose, and purine metabolism, which are known to play roles in systemic inflammation and immune regulation.^7^, ^8^, ^9^ Xie et al.^10^ identified that metabolites such as adenosine, citrulline, 4-guanidinobutyric acid, and linoleic acid may differentiate CRSwNP subtypes. Li et al.^11^ found that associations between altered fatty acid metabolism, uric acid accumulation and inflammatory features in sinonasal tissues of patients with CRSwNP. Furthermore, epidemiological studies have shown that the prevalence of CRSwNP is significantly higher in individuals with diabetes mellitus, and elevated fasting blood glucose levels increase the risk of CRS recurrence,^12^^,^^13^ suggesting a potential link between metabolic status and CRS at the population level. However, these findings are largely derived from subtype-specific or tissue-based investigations.

Despite these advances, population-based evidence regarding the association between serum metabolic markers and CRS remains underexplored. In this study, we aimed to epidemiologically examine the associations between serum lipid profiles (low-density lipoprotein cholesterol, LDL-C; triglycerides, TG; high-density lipoprotein cholesterol, HDL-C; Total Cholesterol, TC), purine metabolism (serum Uric Acid, UA), glucose metabolism (Fasting Blood Glucose, FBG), and CRS prevalence, utilizing data from the Korean National Health and Nutrition Examination Survey (KNHANES). In addition, social and environmental determinants may further influence susceptibility to CRS. Differences in educational and socioeconomic status, residential environment, dietary habits, metabolic health, exposure to air pollutants and allergens, and health awareness have all been linked to upper airway inflammatory diseases.^14^^,^^15^ Therefore, we incorporated education level and residential area as potential effect modifiers to examine whether social factors alter the relationship between metabolic status and CRS.

## Methods

### Study design and participants

**Fig. 1:**
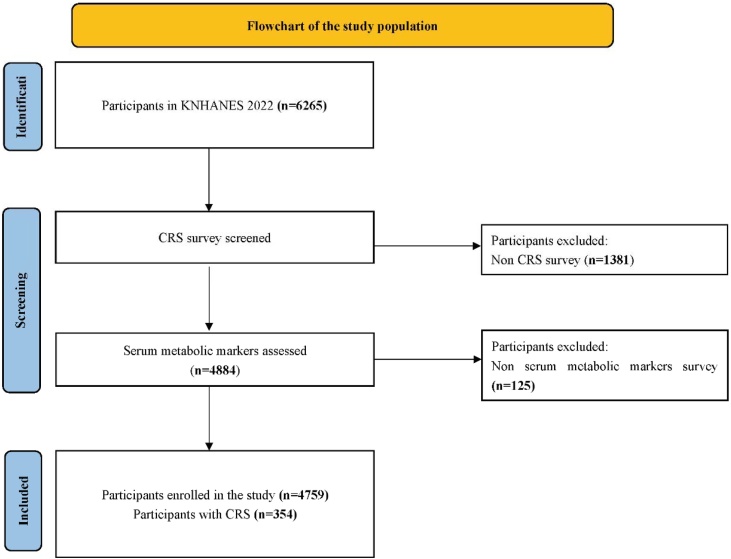
Flowchart of the study population.

Our study used data collected from the KNHANES (2022 survey). KNHANES is a government-approved annual statistical survey managed by the Korea Centers for Disease Control and Prevention (KCDC), designed to evaluate health-related behaviors, health status, and nutritional status of adults in Korea.^16^ A total of 6,265 participants were initially included in this study. After excluding 1,381 participants who did not undergo the CRS survey and 125 participants without blood chemistry data, a final cohort of 4,759 participants was included for this study (Fig. 1).

### Definitions of CRS

In clinical practice, CRS is typically defined based on persistent sinonasal symptoms lasting for at least 12-weeks together with objective confirmation, such as nasal endoscopy or computed tomography findings. The diagnosis of CRS was determined through health interviews. Specifically, participants who answered “yes” to the question, “Have you been diagnosed with CRS by a doctor?” were considered to have a confirmed diagnosis. Subsequently, participants were categorized into non-CRS and CRS groups accordingly. As KNHANES does not include objective diagnostic assessments such as nasal endoscopy or computed tomography, no distinction was possible regarding CRS phenotype (e.g., CRSwNP vs. CRSsNP), disease severity, or disease activity. Accordingly, CRS status in the present study reflects self-reported, physician-diagnosed CRS at the population level, which may have resulted in outcome misclassification.

### Measurement of serum metabolic markers

Blood samples were obtained from participants following an overnight fasting period of at least 8 -hs. The samples were immediately centrifuged and stored at refrigerated temperatures before being transported to a central testing facility, where analysis was conducted within 24 -hs. Serum levels of TC, TG, and HDL-C were measured using the Hitachi 7600 Automated Analyzer (Hitachi, Tokyo, Japan). LDL-C concentrations were calculated using the Friedewald formula; however, when serum TG levels were ≥ 400 mg/dL, direct measurement was performed using a commercially available kit (Cholestest® LDL; Sekisui Medical, Tokyo, Japan). Serum uric acid levels were determined by the colorimetric method using the Hitachi 7600-210 Automated Analyzer (Hitachi Healthcare, Tokyo, Japan). FBG levels were measured using the hexokinase UV method with the Hitachi 7600-210 Automated Analyzer (Hitachi, Tokyo, Japan).

In line with the National Cholesterol Education Program Adult Treatment Panel III guidelines,^17^ high TC was characterized as a level ≥240 mg/dL or the use of lipid-lowering medication; high LDL-C was identified as a level ≥160 mg/dL or the use of lipid-lowering medication; high TG was labeled as a level ≥200 mg/dL; and low HDL-C was defined as a level ≤40 mg/dL. Hyperuricemia was defined as a serum UA level ≥6.0 mg/dL in females and ≥7.0 mg/dL in males.^18^ In accordance with the American Diabetes Association guidelines, hyperglycemia was identified as a FBG level ≥126.0 mg/dL.^19^

### Confounders assessment

Demographic characteristics in this study encompassed sex (male/female), age, educational level (college or above, high school graduate, less than high school), household income (divided into quartiles), residential area (urban/rural), and occupational category. Lifestyle assessments included alcohol consumption (less than twice a week or twice or more a week) and smoking (fewer or more than five packs of cigarettes in a lifetime). The health-related variables considered Body Mass Index (BMI), computed as the ratio of weight (kg) to the height in meters squared (m^2^), with a cutoff point of 25, categorizing participants into non-obese (BMI < 25) and obese groups (BMI ≥ 25). Furthermore, allergic rhinitis and asthma were also included as potential confounding factors in the study.

### Statistical analysis

Data analysis in this survey was performed using IBM SPSS software (version 26.0 for Mac). Following the statistical guidelines provided by the KCDC, we employed complex sample analysis procedures to optimize the use of sampling weights, stratification, and clustering variables from the KNHANES, ensuring nationally representative estimates. Continuous variables are shown as mean ± standard deviation, and categorical variables are expressed as frequencies and percentages. To evaluate the relationship between serum metabolic biomarkers and CRS, we performed complex sample multivariate logistic regression analysis to estimate the Odds Ratios (OR) and associated 95% Confidence Intervals (95% CI), while accounting for potential confounders, including gender, age, household income, educational level, occupation, place of residence, alcohol consumption, smoking status, BMI, allergic rhinitis, and asthma. To assess potential multicollinearity among independent variables, Variance Inflation Factors (VIFs) were calculated, with all values < 10 indicating no evidence of multicollinearity. Statistical significance was determined at a p-value < 0.05.

## Results

### Demographic information of study participants

This study successfully included 4759 participants with an average age of 53.05 ± 17.12 years old. In terms of gender distribution, males accounted for 43.2% and females for 56.8%. The majority of participants exhibited lifestyle habits characterized by a lifetime consumption of fewer than five packs of cigarettes (63.5%), drinking alcohol less than twice a week (77.4%), a BMI < 25 kg/m^2^ (64.1%), living in urban areas (78.7%). In addition, 92.6% had no history of allergic rhinitis, and 96.8% had no history of asthma. The overall prevalence of CRS in this study was 7.4%. After applying sampling weights from the complex survey design, this corresponds to an estimated 2.9 million individuals with CRS nationwide in South Korea (Table 1).

**Table 1:** Characteristics of the study population according to chronic rhinosinusitis status.

Characteristics	n	Weighted N in millions	Overall	No chronic rhinosinusitis^a^	Chronic rhinosinusitis^b^
Age (years), Mean (SE)	4759	39.2	53.05 ± 17.12	53.50 ± 17.10	47.34 ± 16.33
Sex, n (%)					
Male	2055	16.9	43.2%	1923 (40.4%)	132 (2.8%)
Female	2704	22.3	56.8%	2482 (52.1%)	222 (4.7%)
Educational level, n (%)					
Below high school	1270	10.5	26.7%	1216 (25.6%)	55 (1.2%)
High school graduate	1553	12.8	32.7%	1455 (30.6%)	98 (2.1%)
College degree or above	1926	15.9	40.6%	1725 (36.2%)	201 (4.2%)
Residence, n (%)					
Urban	3747	30.9	78.7%	3438 (72.2%)	309 (6.5%)
Rural	1012	8.3	21.3%	967 (20.3%)	45 (1.0%)
Smoking, n (%)					
< 5 pack in a lifetime	3016	24.9	63.5%	2785 (58.6%)	231 (4.9%)
≥ 5 pack in a lifetime	1732	14.3	36.5%	1610 (33.9%)	122 (2.6%)
Alcohol intake, n (%)					
< 2 times a week	3294	30.3	77.4%	3029 (71.2%)	265 (6.2%)
≥ 2 times a week	963	8.9	22.6%	898 (21.1%)	65 (1.5%)
BMI (Kg/m^2^), n (%)					
< 25	3002	25.1	64.1%	2774 (59.2%)	228 (4.9%)
≥ 25	1684	14.1	35.9%	1558 (33.2%)	126 (2.7%)
Asthma, n (%)					
No	4608	38.0	96.8%	4277 (89.8%)	331 (7.0%)
Yes	151	1.2	3.2%	128 (2.7%)	23 (0.5%)
Allergic rhinitis, n (%)					
No	4405	36.3	92.6%	3763 (79.1%)	642 (13.4%)
Yes	354	2.9	7.4%	188 (4.0%)	166 (3.5%)
Metabolic serum markers					
**High FBG**					
No	4377	36.1	92.0%	4041 (84.9%)	336 (7.1%)
Yes	382	3.1	8.0%	364 (87.6)	18 (0.4%)
High UA					
No	4202	34.6	88.3%	3898 (81.9%)	304 (6.4%)
Yes	557	4.6	11.7%	507 (10.7%)	50 (1.0%)
High TC					
No	4287	35.3	90.1%	3961 (83.2%)	326 (6.9%)
Yes	472	3.9	9.9%	444 (9.3%)	28 (0.6%)
High TG					
No	4152	34.2	87.2%	3837 (80.6%)	315 (6.6%)
Yes	607	5.0	12.8%	568 (11.9%)	39 (0.8%)
High LDL-C					
No	4260	35.1	89.5%	3932 (82.6%)	328 (6.9%)
Yes	499	4.1	10.5%	473 (9.9%)	26 (0.5%)
High HDL-C					
No	4170	34.3	87.6%	3844 (80.8%)	326 (6.8%)
Yes	589	4.9	12.4%	561 (11.8%)	28 (0.6%))

BMI, Body Mass Index; UA, Uric Acid; TC, Total Cholesterol; TG, Triglycerides; HDL-C, High Density Lipoprotein Cholesterol; LDL-C, Low Density Lipoprotein Cholesterol; FBG, Fasting Blood Glucose.

a No chronic rhinosinusitis: n = 4405; Weighted N in millions = 36.3.

b Chronic rhinosinusitis: n = 354; Weighted N in millions = 2.9.

### Multivariate analysis

**Fig. 2:**
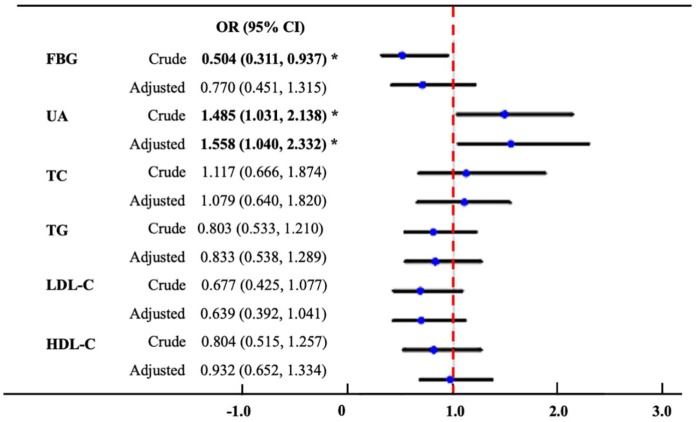
Logistic analysis showing the association between serum metabolic markers and chronic rhinosinusitis. UA, Uric Acid; TC, Total Cholesterol; TG, Triglycerides; HDL-C, High Density Lipoprotein Cholesterol; LDL-C, Low Density Lipoprotein Cholesterol; FBG, Fasting Blood Glucose.

After controlling for all potential confounders, logistic regression analysis indicated a significant association between serum UA and the prevalence of CRS. Specifically, there was a notable correlation between serum UA levels and the presence of CRS (OR = 1.558, 95% CI 1.040, 2.332, p = 0.020). However, no marked association was identified between serum markers of lipid metabolism and glucose metabolism and the prevalence of CRS (Fig. 2).

### Subgroup analysis with different characteristics

Following subgroup analysis of confounding factors with p < 0.05 in the multivariate regression analysis (Table 2), an association between high levels of UA and an elevated likelihood of CRS was observed, particularly among urban residents (OR = 1.635, 95% CI 1.085, 2.463, p = 0.019) (Table 3).

**Table 2:** Odds ratios of potential confounding factors in multiple logistic regression analysis for CRS.

Confounding factors	OR (95% CI)	p-value
Age	**0.997 (0.987, 1.006)**^a^	**0.507**
Sex	**1.297 (0.953, 1.767)**	**0.098**
BMI	0.986 (0.956, 1.016)	0.342
Residence	**0.640 (0.428, 0.957)**^a^	**0.030**
Education level	**1.248 (1.026, 1.519)**^a^	**0.027**
Occupation	0.991 (0.932, 1.053)	0.763
House income	0.973 (0.860, 1.100)	0.659
Smoking	0.964 (0.783, 1.185)	0.724
Alcohol intake	0.994 (0.926, 1.066)	0.858
Asthma	**1.774 (0.920, 3.418)**	**0.086**
Allergic rhinitis	**4.547 (3.468, 5.962)**^b^	**0.000**

OR, Odds Ratio; BMI, Body Mass Index; CRS, Chronic Rhinosinusitis.

a p < 0.05;

b p < 0.001.

**Table 3:** Subgroup analysis for the association between serum UA and CRS patients.

CRS	Education level			
OR (95% CI)	Below high school	High school graduate	College degree or higher	
High UA	1.032 (0.281, 3.792)	0.997 (0.336, 2.962)	0.914 (0.420, 1.990)	
Yes vs. No	p = 0.962	p = 0.996	p = 0.821	

CRS	Residence		Allergic rhinitis	
OR (95% CI)	Urban	Rural	No	Yes
High UA	**1.635 (1.085, 2.463)**^a^	0.787 (0.228, 2.719)	1.400 (0.832, 2.354)	1.729 (0.951, 3.144)
Yes vs. No	**p = 0.019**	p = 0.704	p = 0.204	p = 0.072

UA, Uric Acid; CRS, Chronic Rhinosinusitis.

a p < 0.05.

## Discussion

In this study, we utilized data from a nationwide cross-sectional survey and found a significant correlation between higher serum UA levels and the presence of CRS, particularly among individuals living in urban areas. However, no remarkable association was found between serum indices of lipid metabolism and glucose metabolism and the prevalence of CRS. To our knowledge, this is the first large-scale cross-sectional study to examine the association between serum lipid metabolism, glucose metabolism, purine metabolism, and the prevalence of CRS.

Previous metabolomics studies in CRS have identified alterations in lipid mediators, such as fatty acids, linoleic acid, and maresin, which affect the inflammatory process and disease severity in CRS.^10^^,^^20^ In addition, in two population-based cross-sectional studies, Wee et al.^21^ observed that a history of dyslipidemia was associated with a higher risk of CRS. With respect to glucose metabolism, Chen et al.^22^ demonstrated that elevated glucose levels in nasal secretions promote glucose uptake and glycolysis in epithelial cells, enhancing the pro-inflammatory responses of CRS epithelial cells, indicating a crucial role of glycolysis in CRS pathogenesis. In contrast, Lee et al.^23^ found no significant difference in the overall prevalence of CRS between individuals with and without metabolic syndrome. These inconsistencies may be attributed to variations in study populations, as metabolic syndrome encompasses abnormalities in waist circumference, blood pressure, and FBG, in addition to dyslipidemia. Regarding purine metabolism, recent evidence highlights a potential role for UA in CRS. Xie et al.^24^ found significantly higher serum UA levels in patients with recurrent CRS in comparison to non-recurrent patients, while FBG levels did not differ significantly, indicating a correlation between increased serum UA levels and elevated risk of postoperative recurrence in CRS. Similarly, Jiang et al.^25^ found that hyperuricemia independently predicted recurrence in Chinese patients with CRS. In our study, we observed that elevated UA levels were associated with the presence of CRS, whereas other metabolic parameters showed no significant association. Taken together, these findings support an epidemiological association between serum UA levels and CRS at the population level. Therefore, further research and validation are necessary to identify suitable metabolic biomarkers that may be associated with CRS prevalence and phenotypic heterogeneity.

The biological mechanisms linking elevated serum UA levels to CRS are not yet fully understood. Importantly, most mechanistic evidence regarding UA and airway inflammation is derived from experimental models or tissue-based studies, rather than from population-level analyses. Previous studies have suggested that UA may influence inflammatory pathways, including NLRP3 inflammasome activation, epithelial-mesenchymal transition, and immune cell recruitment.^26^^,^^27^ For example, experimental studies have shown that elevated UA levels may promote epithelial barrier dysfunction and inflammatory responses under specific conditions.^28^^,^^29^ However, these mechanistic findings cannot be directly extrapolated to infer causality in the present population-based, cross-sectional study. Instead, they provide a possible biological context for interpreting the observed epidemiological association between serum UA levels and CRS.

Exploratory subgroup analyses further revealed heterogeneity across sociogeographic factors. The association between elevated UA levels and CRS appeared stronger among individuals living in urban areas. This trend aligns with prior evidence identifying urban residence as a risk factor for CRS, potentially due to greater exposure to air pollutants, industrial emissions, and allergenic particulates.^30^^,^^31^ Another plausible explanation is differential healthcare utilization, as urban populations generally possess better access to otolaryngologic services and may seek medical evaluation for sinonasal symptoms more readily.^32^ Therefore, the stronger UA-CRS association observed in urban settings may partly reflect increased disease detection or diagnostic opportunities rather than a purely biological effect, highlighting the potential impact of diagnostic bias and differential access to healthcare in population-based CRS research. In contrast, educational attainment did not modify the association between UA levels and CRS in our study. This finding differs from Lee et al., who reported a higher CRS prevalence among individuals with higher education levels, possibly reflecting greater health awareness and symptom reporting.^15^ The lack of effect modification in our cohort may indicate that education-related differences in health literacy or care-seeking behavior exert limited influence on UA-related CRS susceptibility. Alternatively, the absence of significant interaction may be attributable to the relatively coarse categorization of education level, potential residual confounding, or insufficient statistical power within subgroups. These considerations highlight the need for future research employing richer sociobehavioral measures to better elucidate how educational or behavioral factors shape CRS detection and metabolic risk profiles.

The present study has several limitations that should be considered. Firstly, the study used a cross-sectional design, which restricts the ability to determine a causal relationship between serum metabolic markers and the prevalence of CRS. Secondly, the CRS diagnosis relied on self-reported information provided by the participants, without objective confirmation such as nasal endoscopy or imaging, which may have introduced recall bias, outcome misclassification, and diagnostic bias related to differential healthcare access or healthcare-seeking behavior. Thirdly, the study did not include detailed clinical information on the severity, endotypes, or subtypes of CRS, which could affect the relationship between metabolic markers and CRS. Additionally, information on environmental exposure, such as air pollution, was unavailable in the KNHANES dataset, which might have limited our ability to fully adjust for all potential confounders. Finally, the study focused on a limited number of metabolic markers. Other potentially relevant markers of lipid, glucose, and purine metabolism were not included, which could have influenced the results. Despite these limitations, our study provides population-based epidemiological evidence of an association between serum uric acid levels and CRS prevalence in a large, nationally representative sample.

## Conclusions

In this population-based cross-sectional study, we observed a significant association between higher serum UA levels and the presence of CRS, particularly among urban residents. These findings suggest that serum UA may serve as an associated biomarker of CRS at the population level, rather than a causal determinant. Future longitudinal studies are warranted to evaluate these associations and to explore potential causal pathways.

## ORCID ID

Lin Wang: 0000-0002-6710-2971

Jisheng Zhang: 0000-0001-9674-8483

Xudong Yan: 0000-0001-5302-2850

## Authors' contributions

Han Chen: Conceptualization; methodology; validation; formal analysis; writing-original draft.

Lin Wang: Software; validation; formal analysis; investigation; writing-review & editing.

Jisheng Zhang: Software; validation; formal analysis; investigation; writing-review & editing.

Xudong Yan: Investigation; data curation; writing-review & editing.

Longgang Yu: Conceptualization, methodology, writing-review & editing, supervision.

Yan Jiang: Conceptualization; methodology; writing-review & editing; supervision; funding acquisition.

## Fundings

This work was supported by grants from the Program for Natural Science Foundation of Shandong Province (ZR2023MH027), Medicine and Health Science Technology Development Program of Shandong Province (202207010780), and Natural Science Foundation of Qingdao Municipality (23-2-1-199-zyyd-jch), National Natural Science Foundation of China (82471140).

## Data availability statement

The datasets analyzed in this study are from the KNHANES 2022, which are publicly available and can be downloaded from the KNHANES website: https://knhanes.kdca.go.kr/knhanes/main.do.

## Declaration of competing interest

The authors declare no conflicts of interests.
